# The Liver X Receptor Agonist TO901317 Ameliorates Behavioral Deficits in Two Mouse Models of Autism

**DOI:** 10.3389/fncel.2019.00213

**Published:** 2019-05-14

**Authors:** Yulong Cai, Hongyu Zhong, Xin Li, Rui Xiao, Lian Wang, Xiaotang Fan

**Affiliations:** Department of Developmental Neuropsychology, School of Psychology, Third Military Medical University, Chongqing, China

**Keywords:** autism, neurogenesis, sociability, TO901317, repetitive behavior

## Abstract

Autism spectrum disorder (ASD) is a developmental disability characterized by social deficits and repetitive stereotyped behaviors. There are currently no drugs available for the treatment of the core symptoms of ASD, suggesting an urgent need for new therapeutic strategies. The neurobiology of autism is complex, but emerging research indicates that defects in hippocampal neurogenesis are associated with ASD in both humans and mouse models of ASD, leading to the suggestion that restoring neurogenesis may be a novel therapeutic approach for ASD. Here, we found that postnatal treatment with TO901317 (TO), a potent liver X receptor (LXR) agonist, typically activated LXRβ and its target genes in the hippocampus, and alleviated the social deficits and stereotypical behaviors in BTBR T+ tf/J (BTBR) and valproic acid (VPA)-induced mouse models. In addition, we further confirmed that TO postnatal treatment also rescued the inhibition of adult hippocampal neurogenesis in these two models. In summary, our study suggests that LXR agonist targeting hippocampal neurogenesis may represent a novel potential therapy for ASD.

## Introduction

Autism spectrum disorder (ASD) is a neurodevelopmental disability characterized by impaired sociability, as well as restricted, repetitive patterns of interests and behaviors (Rubenstein and Merzenich, 2003; Currais et al., 2016; Chao et al., 2018). Over the past decades, the incidence of ASD has increased dramatically, reaching 1 in 59 children (Baio et al., 2018). Existing data have revealed that families with autistic children are faced with high levels of economic pressures without any effective therapy (Chan et al., 2018). Thus, there is an urgent need to determine the etiology of ASD and search for an effective treatment. Despite a growing body of ASD studies, the mechanism underlying this disorder still remains poorly understood. At present, no consensus has been reached regarding the neurobiological mechanisms of ASD, and many factors have been shown to be involved in the pathogenesis of ASD, such as synapse dysfunctions, neuroimmunity, oxidative stress, and neurogenesis (Pardo and Eberhart, 2007; Jyonouchi et al., 2008; Carpita et al., 2018).

The hippocampus is a brain region with a high level of plasticity throughout the lifespan, generating new neurons within the subgranular zone (SGZ) of the dentate gyrus (DG) (Xiao et al., 2016; Nam et al., 2017). It demonstrates that new neurons produced in the hippocampus play a critical role in mediating emotion and cognition (Juliandi et al., 2015). Recently, amount of evidences have indicated that deficits in hippocampal neurogenesis are associated with ASD both in humans and in mouse models of ASD (Escamilla et al., 2017; Abookasis et al., 2018). Studies highlight that hippocampal plasticity contributes to social behavior. It seemed that hippocampal neurogenesis triggering plasticity could restore normal behavioral phenotype.

The liver X receptor (LXR), LXRα, and LXRβ, belong to the large family of ligand-activated transcription factors (Mohan and Heyman, 2003; Houck et al., 2004). It has been demonstrated that LXRβ is ubiquitously expressed, and especially abundant in the central nervous system (CNS) and endocrine system, while LXRα expression is restricted to tissues rich in lipid metabolism such as intestine, kidney, liver, and spleen (Peet et al., 1998; Repa and Mangelsdorf, 2000; Whitney et al., 2002). Our previous study confirmed LXRβ was strongly expressed in the hippocampus and involved in the DG formation and neurogenesis. Deletion LXRβ in mice showed decreased neurogenesis in the postnatal and adult hippocampus, as well as autistic-like behaviors, including impaired sociability and increased repetitive self-grooming (Cai et al., 2018). These suggest that LXRβ activation may alleviate autistic-like behaviors by improving neurogenesis.

With the increasing number of animal models, we have a powerful tool to discover the mechanism and treatments of diseases under well-controlled conditions. In recent years, considerable efforts have been made to establish a reliable rodent model that can represent the abnormal behaviors observed in ASD. Among the many models available, the inbred BTBR T+ Itpr3tf/J (BTBR) mouse strain is a very well-built mouse model of autism, which displays increased repetitive self-grooming and marble burying, increased cognitive rigidity, impaired sociability, and abnormal vocalizations (Silverman et al., 2010; Stephenson et al., 2011; Currais et al., 2016). Exposure of mice to valproic acid (VPA) is an another valid ASD model used in preclinical experiments. A single prenatal (embryonic day 13) or neonatal (postnatal day 14) injection of VPA can induce autistic behaviors, including repetitive/stereotypic-like activity, impaired sociability, decreased nociceptive reactivity, and abnormal communication, in mice or rats (Yochum et al., 2008). A growing body of data has identified reduced hippocampal neurogenesis in the adult brain of VPA-exposed and BTBR mice (Yochum et al., 2008; Stephenson et al., 2011; Juliandi et al., 2015). Several studies demonstrated that treatments promoting neurogenesis could improve sociability and decrease repetitive behaviors in these ASD models (Currais et al., 2016; Gobshtis et al., 2017).

In the present study, the role of LXR agonist TO in alleviating autistic behavior in the two ASD mouse models was investigated. Early postnatal mice received TO intraperitoneal injection, the behavior and hippocampal neurogenesis were analyzed. The results showed that TO treatment could increase hippocampal precursor proliferation and alleviate the social deficits in the BTBR and VPA-induced mouse models of ASD, which have shown decreased DG neurogenesis. Our finding indicates that drug development targeting neurogenesis may be an effective method for ASD treatment.

## Materials and Methods

### Animals

All mice were maintained in the Animal Facility of the Third Military Medical University with controlled temperature, standard 12 h light/dark cycle and mouse chow and water provided *ad libitum*. The C57BL/6J (B6) and BTBR mice breeding pairs were provided by the Third Military Medical University and the Model Animal Research Center of Nanjing University (Nanjing, China), respectively. Only the male pups of the B6 and BTBR mice were used in our experiment. All experimental procedures were approved by the Third Military Medical University and were also performed in accordance with the Guidelines for Animal Care and Use. Every effort was made to restrict the use of animals to as few as possible in our experiment.

### Drug Treatment

#### TO Treatment in BTBR Mice

**FIGURE 1:**
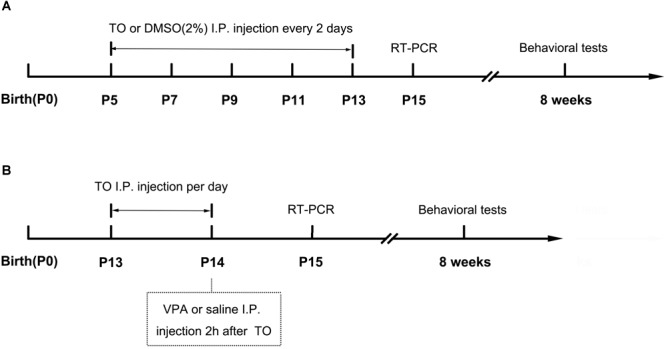
Schematic diagram of the experimental procedures. **(A)** Neonatal male mice (B6 or BTBR) were injected with TO (50 mg/kg) or 2% DMSO from PD 7 to PD 13 every 2 days. The RT-PCR was conducted on PD 15. The behavioral tests were conducted at the age of 8 weeks. **(B)** Neonatal male mice (B6) were injected with TO (50 mg/kg) or 2% DMSO on PD 13 and PD 14, once per day, and then injected with VPA (400 mg/kg) or 0.9% NaCl 2 h after TO injection on PD 14. The RT-PCR was conducted on PD 15. The behavioral tests were conducted at the age of 8 weeks.

The day of birth was designated as postnatal day 0 (PD 0). B6 male mice were used as the normal controls. On PD 5, male pups were randomly divided into the following four groups: (1) 2% dimethyl sulfoxide (DMSO) (B6), (2) TO (B6+TO), (3) 2% DMSO (BTBR), or (4) TO (BTBR+TO). TO was dissolved in 100% DMSO and diluted with PBS to a final concentration of 2% before intraperitoneal (i.p.) injection to the pups (50 mg/kg) on PD 5, PD 7, PD 9, PD 11, and PD 13 every 2 days (Figure 1A). Behavior tests were conducted at the age of 8 weeks.

#### TO Treatment in VPA-Exposed Mice

The day of birth was designated as PD 0. On PD 13, B6 male mice were randomly divided into the following four groups: (1) 2% DMSO and normal saline (0.9% NaCl) (B6), (2) TO and normal saline (0.9% NaCl) (TO), (3) 2% DMSO and 400 mg/kg VPA (Sigma) (VPA), or (4) pretreatment of TO followed by VPA (VPA+TO). TO was dissolved in 100% DMSO and diluted with PBS to a final concentration of 2% before intraperitoneal (i.p.) injection to the pups (50 mg/kg) on PD 13 and PD 14, once per day. The control groups received an equivalent dose of the vehicle DMSO. On PD 14, the VPA and VPA+TO (2 h after TO injection) received 400 mg/kg VPA intraperitoneally (Yochum et al., 2008). The controls were given the same volume of sterile saline (Figure 1B). Behavior tests were conducted at the age of 8 weeks.

### Behavioral Tests

Both the three chambered social approaches and self-grooming tests were conducted with B6 and BTBR male mice at the age of 8 weeks during daytime of the circadian cycle. Mice were placed into the experimental room for at least 30 min prior to each test to adjust to the environment. Eight mice per group were utilized for analysis.

### Three-Chambered Social Approach

The three-chambered social approach test was performed in a rectangular apparatus (40 cm × 60 cm × 22 cm, divided into three equal parts) based on a previous protocol (Yang et al., 2011). The test consisted of a habituation phase and two testing phases, including sociability and social novelty recognition. During the first 10-min session, the subject mouse was habituated in the center chamber and had free access to the three-chambered box. During the sociability session, the subject mouse was allowed to explore the entire box for another 10 min with a novel male mouse (S1) and a novel object (O) introduced to the side chambers. After that, a third 10-min session was performed to assess the preference of social novelty, in which the object was replaced by an unfamiliar male mouse (S2) at the previous position. Seventy percent of ethanol and water was used to clean the whole arena between tests. The total time in each chamber for each phase was detected by using Ethovision XT 11.5. The preference index, which indicates the numerical time difference between chambers (S1 vs. O or S2 vs. S1) divided by total time in side chambers.

### Self-Grooming

Mice were individually and gently placed into a standard mouse cage. The tests consisted of a 10-min habituation phase and a 10-min test phase with a video camera 15 cm away from the cage. The total time of self-grooming and the number of rearings in the test phase were counted with the researcher blinded to the treatment. Seventy percent of ethanol and water was used to clean the whole arena between tests (Silverman et al., 2015).

### BrdU Labeling

To assess cell proliferation in the hippocampus, the mice were injected with bromodeoxyuridine (BrdU, 100 mg/kg; Sigma-Aldrich) intraperitoneally every 12 h for 3 times before anesthesia.

### Immunohistochemistry and Immunofluorescence

Based on our previous experiment (Liu et al., 2016), the mice were perfused transcardially with 0.9% saline followed by 4% paraformaldehyde (PFA) after deep anesthesia with pentobarbital. The brain was collected and dehydrated completely in 30% sucrose solution with 4% paraformaldehyde at 4°C. Brains were sliced coronally (30 μm thick) and stored at -20°C in cryoprotectant solution. Immunostaining was performed according to our previous method. In short, the brain sections were incubated with a rabbit anti-doublecortin (DCX) (1:1000, Cell Signaling) primary antibody in 1% bovine serum albumin (BSA) (12 h, 4°C). For BrdU staining, the sections were pretreated with 2 N HCl for 30 min at 37°C and then incubated with mouse anti-BrdU (1:500, BD Biosciences). After that, the sections were incubated with biotin-conjugated secondary antibody (1:200, goat anti-rabbit; Invitrogen) or Cy3 (1:500; donkey anti-mouse; Jackson ImmunoResearch) (2 h, 37°C), then treated with avidin-biotin peroxidase complex (Dako) for immunohistochemistry or 4′, 6-diamidino-2-phenylindole (DAPI, Beyotime, China) for immunofluorescence. The BrdU- or DCX-labeled cells were observed and photographed with a Zeiss (Oberkochen, Germany) Axivert microscope equipped with a Zeiss AxioCam digital color camera connected to the Zeiss AxioVision 3.0 system.

### Cell Counting and Unbiased Stereology

According to our previous method (Liu et al., 2016), every 10th section (30 μm thick) through the rostrocaudal extent of the DG was selected to evaluate the number of BrdU^+^ or DCX^+^ cells in the granule cell layer (GCL) plus SGZ. Stereological cell quantification was used as described previously to calculate the total number of the two kinds of cells in the DG. Three mice per group were utilized for analysis.

### Real-Time PCR

The brains were isolated on PD 15 and then used for RT-PCR as our previous method (He et al., 2017). Briefly, total RNA was extracted by an RNeasy kit (CWBIO, Cat.CW05815, China) based upon the instructions from the manufacturer. Then, total RNA (approximately 1–2 μg per 20 μl reaction) was reverse transcribed to cDNA using the PrimeScript RT Reagent Kit (Takara) after the concentration was detected qualified by a spectrophotometric instrument (NanoDrop). The CFX96 Real-Time PCR system (Bio-Rad) was used to conduct the RT-PCR analysis. The primer sequences of ABCA1, ABCG1, LXRß, and GAPDH were designed as follows: mouse LXRα, forward: 5′-TCCATCAACCACCCCCACGAC-3′ reverse: 5′-CAGCCAGAAAACACCCAACCT-3′; mouse LXRß, forward: 5′-TCGCCATCAACATCTTCTCAG-3′, reverse: 5′-GTGTGGTAGGCTGAGGTGTAA-3′; mouse ABCA1, forward: 5′-GGGTGAACGAGTTTCGGTATG-3′, reverse: 5′-CTGAAGATGCTTGGCTTTGCT-3′; mouse ABCG1, forward: 5′-AGAAAGGATGAAGGCAGACGG-3′ reverse: 5′-TGCTGGGTTGTGGTAGGTAGGG-3′; and mouse GAPDH, forward: 5′-AGGTCGGTGTGAACGGATTTG-3′, and reverse: 5′-TGTAGACCATGTAGTTGAGGTCA-3′. The relative expression levels were normalized to GAPDH and analyzed using the 2^-ΔΔCt^ method.

### Statistical Analyses

The data are presented as the mean ± SEM. The results of self-grooming, real-time PCR and immunostaining were analyzed using two-way ANOVA. The data of the three-chambered social approach task were analyzed with two-way ANOVA, repeated measures ANOVA and paired *t*-test (Silverman et al., 2015; Zhang et al., 2018). Significant effects were evaluated with a least significant difference (LSD) *post hoc* test. The statistical significance was set at *p* < 0.05.

## Results

### TO Upregulated the Expression of LXRß and Its Target Genes, ABCA1, and ABCG1, in the Hippocampus

**FIGURE 2:**
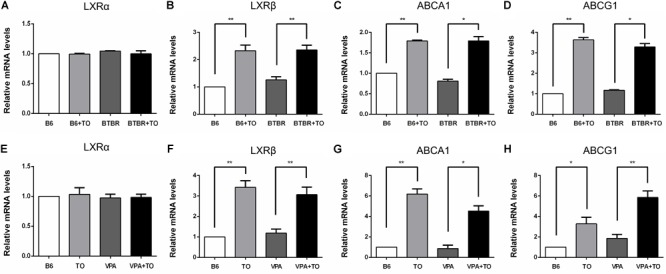
TO treatment upregulated the relative mRNA levels of LXRß and its target genes, ABCA1 and ABCG1, in the hippocampus. **(A,E)** TO treatment did not influence the expression of LXRα among the mice. **(B–D)** TO treatment increased the expression of LXRß [two-way ANOVA: drug effect: *F*(1,8) = 69.296, *p* < 0.01; genotype effect: *F*(1,8) = 0.997, *p* > 0.05; drug × genotype effect: *F*(1,8) = 0.618, *p* > 0.05, followed by LSD *post hoc* test] and its target genes, ABCA1 [two-way ANOVA: drug effect: *F*(1,8) = 234.636, *p* < 0.01; genotype effect: *F*(1,8) = 2.705, *p* > 0.05; drug × genotype effect: *F*(1,8) = 2.706, *p* > 0.05, followed by LSD *post hoc* test] and ABCG1 [two-way ANOVA: drug effect: *F*(1,8) = 566.361, *p* < 0.01; genotype effect: *F*(1,8) = 0.758, *p* > 0.05; drug × genotype effect: *F*(1,8) = 6.819, *p* < 0.05, followed by LSD *post hoc* test] in B6 and BTBR mice. **(F–H)** TO treatment upregulated the expression of LXRß [two-way ANOVA: TO effect: *F*(1,8) = 67.092, *p* < 0.01; VPA effect: *F*(1,8) = 0.097, *p* > 0.05; TO × VPA effect: *F*(1,8) = 1.047, *p* > 0.05, followed by LSD *post hoc* test] and its target genes, ABCA1 [two-way ANOVA: TO effect: *F*(1,8) = 87.464, *p* < 0.01; VPA effect: *F*(1,8) = 12.967, *p* < 0.01; TO × VPA effect: *F*(1,8) = 1.428, *p* > 0.05, followed by LSD *post hoc* test] and ABCG1 [two-way ANOVA: TO effect: *F*(1,8) = 53.395, *p* < 0.01; VPA effect: *F*(1,8) = 9.152, *p* < 0.05; TO × VPA effect: *F*(1,8) = 1.010, *p* > 0.05, followed by LSD *post hoc* test] in the B6 and VPA-exposed mice. Data are presented as the mean ± SEM. *n* = 3. ^∗^*p* < 0.05, ^∗∗^*p* < 0.01.

It has been verified that LXRß is of great importance in DG neurogenesis and hippocampus-related functions (Cai et al., 2018; Sun et al., 2018). In our further experiment, after the intraperitoneal injection of TO, the relative mRNA level of LXRß in the hippocampus was markedly increased in the B6 and BTBR mice compared with that in the same strains treated with saline (Figure 2B). ABCA1 and ABCG1, the target genes of LXRß, were changed similarly to LXRß in the four groups (Figure 2C,D).

Similarly, the relative mRNA level of LXRß in the hippocampus was also upregulated in B6 and VPA-exposed mice compared with that in mice treated with saline (Figure 2F). In accordance with the upregulation of the LXRβ level, the expression of the downstream genes, ABCA1 and ABCG1, was significantly increased (Figure 2G,H). Nevertheless, there was little LXRα expression in the hippocampus of the mice, and TO did not alter the expression of LXRα in any of these mice (Figure 2A,E).

### Early Postnatal TO Treatment Ameliorated the Social Deficits of BTBR Mice in the 3-Chambered Social Approach Task

**FIGURE 3:**
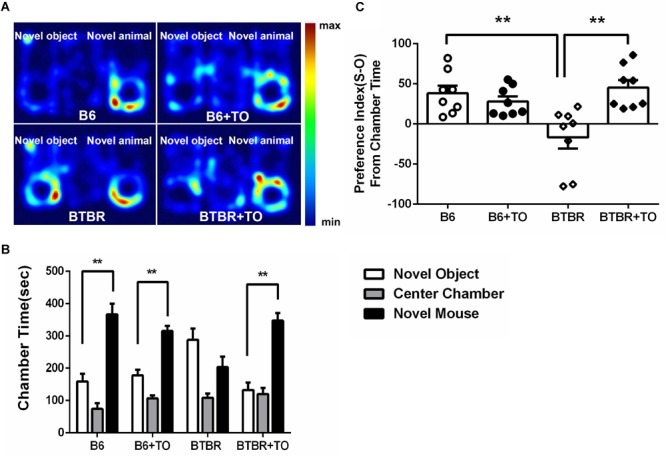
Early postnatal intervention of TO rescued the sociability of BTBR mice. **(A)** Representative heat maps indicate the time distribution of the experimental mice in the sociability test. Warmer colors (red) represent longer stay. **(B)** Saline-treated and TO-treated B6 mice spent more time in the chamber with a novel mouse than with a novel object (paired *t*-test: *p* < 0.01). BTBR mice displayed impaired sociability and showed a preference toward the novel mouse compared with the novel object. Nevertheless, TO pretreatment reversed the preference significantly (paired *t*-test: *p* < 0.01). **(C)** BTBR mice displayed a lower social preference index (S-O/total) than B6 mice, which could be rescued with TO treatment [two-way ANOVA: drug effect: *F*(1,28) = 6.616, *p* < 0.05; genotype effect: *F*(1,28) = 3.551, *p* = 0.070; drug x genotype effect: *F*(1,28) = 12.998, *p* < 0.01, followed by LSD *post hoc* test]. Data are presented as the mean ± SEM. *n* = 8. ^∗∗^*p* < 0.01.

In the three-chambered social approach test, sociability was regarded as spending more time in the chamber with a novel mouse than in the chamber with a novel object; the time spent in the chamber with the novel mouse or the novel object was measured in each group (Figure 3A). B6 mice are extensively social, which is consistent with the observations of B6 mice in our experiment (Figure 3B). There was no clear difference between the saline-treated B6 mice and the TO-treated B6 mice in the sociability test (Figure 3B). However, the TO-treated BTBR mice did show a preference toward the side chamber with the novel mouse compared with the saline-treated BTBR mice that were found to have no bias to the chamber with the novel mouse or the novel object (Figure 3B). In addition, in the habituation session, all mice from the four groups did not show bias toward any of the three chambers. Furthermore, the social preference index was calculated to evaluate the effect of TO pretreatment on sociability deficits. It was suggested that early postnatal TO administration rescued the social deficits in BTBR mice due to the higher social preference index of TO-treated BTBR mice (Figure 3C).

### TO Pretreatment in the Early Postnatal Stage Improved the Social Behavior of VPA-Exposed Mice in the 3-Chambered Social Approach Task

**FIGURE 4:**
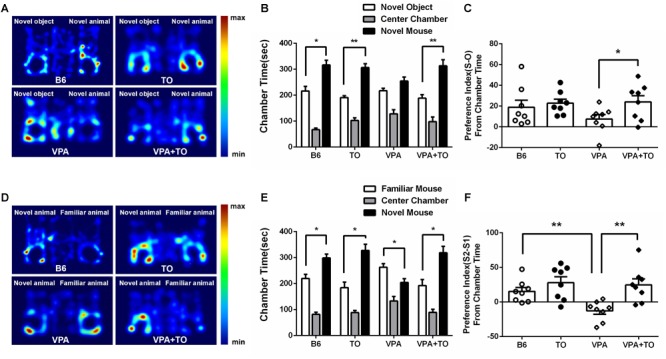
TO pretreatment improved sociability and novel recognition of VPA-exposed mice. **(A)** Representative heat maps indicate the time distribution of the experimental mice in the sociability test. Warmer colors (red) represent longer stay. **(B)** VPA-exposed mice had less interest in the chamber with a novel mouse than did the B6 mice. TO pretreatment greatly alleviated the social deficits of the VPA-exposed mice (paired *t*-test: *p* < 0.01). **(C)** VPA-exposed mice showed a relatively lower preference index (S-O/total) than the B6 mice. TO pretreatment had a clear effect on the improvement of social deficits [two-way ANOVA: TO effect: *F*(1,28) = 3.579, *p* = 0.069; VPA effect: *F*(1,28) = 0.888, *p* > 0.05; TO × VPA effect: *F*(1,28) = 1.347, *p* > 0.05, followed by LSD *post hoc* test]. **(D)** Representative heat maps indicate the time distribution of the experimental mice in the novel recognition test. **(E)** VPA-exposed mice spent more time in the chamber with the familiar mouse (S2) than with the novel mouse (S1) (paired *t*-test: *p* < 0.05). Similarly, TO pretreatment reversed the effect of the VPA exposure (paired *t*-test: *p* < 0.05). **(F)** VPA-exposed mice displayed a significantly lower preference index (S2-S1/total) than the controls; however, TO markedly improved it [two-way ANOVA: TO effect: *F*(1,28) = 11.978, *p* < 0.01; VPA effect: *F*(1,28) = 4.690, *p* < 0.05; TO × VPA effect: *F*(1,28) = 2.963, *p* = 0.096, followed by LSD *post hoc* test]. Data are presented as the mean ± SEM. *n* = 8. ^∗^*p* < 0.05, ^∗∗^*p* < 0.01.

We measured the time spent in side chambers in each group (Figure 4A,D). As stated above, in the sociability test, the B6 mice spent more time in the chamber with the novel mouse than in the chamber with the novel object (Figure 4B). It has been indicated that VPA exposure during early postnatal development could model ASD in mice. Meeting the description of social deficit in this model extensively, the VPA-induced ASD model mice showed more interest in the side chamber with a novel object than the side chamber with a novel mouse (Figure 4B). Nevertheless, following TO treatment, the VPA-exposed mice displayed social behavior comparable to that of controls in the social approach (Figure 4B).

In the social novelty recognition test, the B6 mice spent more time in the chamber with the novel mouse (S2) than in the chamber with the familiar mouse (S1) (Figure 4E), whereas the VPA-exposed mice exhibited the opposite preference to the side chambers, which also indicated that the social recognition of those mice was impaired (Figure 4E). However, TO treatment had a significant effect on the social recognition deficit. As a result, the time VPA-exposed mice spent in side chambers was reversed (Figure 4E).

Taken together, the results showed that the social preference index was influenced by VPA exposure and TO pretreatment. During the second 10-min session, the B6 mice displayed higher sociability, with no significant preference between the chambers with a novel mouse or a novel object, than the VPA-exposed mice (Figure 4C). However, pretreatment with TO increased the social preference index of VPA-exposed mice (Figure 4C), implying that TO markedly improved social deficits in those mice. The conclusions are the same as the social novelty recognition session. There was a significant difference in the social preference index between the saline-treated and VPA-treated mice (Figure 4F). TO pretreatment markedly reversed this effect (Figure 4F).

### Early Postnatal TO Treatment Rescued Spontaneous Behaviors in BTBR Mice and VPA-Exposed Mice

**FIGURE 5:**
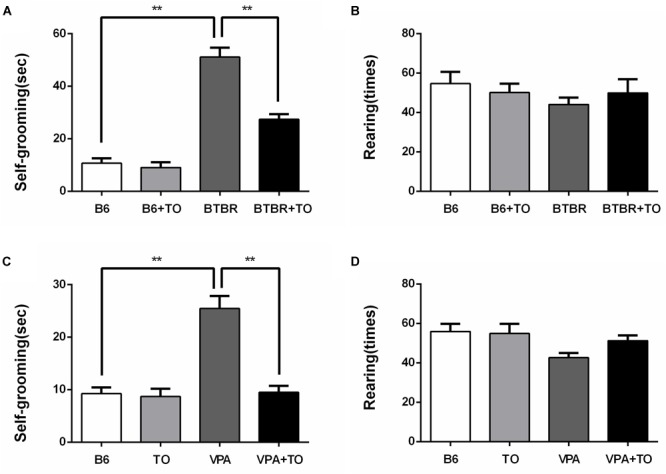
Early postnatal TO treatment rescued spontaneous behaviors in two ASD mouse models (i.e., BTBR and VPA-exposed mice). **(A)** BTBR mice showed high levels of self-grooming compared with B6 mice, and TO alleviated the severe repetitive behavior [two-way ANOVA: drug effect: *F*(1,28) = 26.514, *p* < 0.01; genotype effect: *F*(1,28) = 142.912, *p* < 0.01; drug x genotype effect: *F*(1,28) = 20.111, *p* < 0.01, followed by LSD *post hoc* test]. **(B)** There was no significant difference in the number of rearings among the four groups. **(C)** VPA-exposed mice also displayed high levels of self-grooming compared with the B6 mice, which was also reversed by TO pretreatment [two-way ANOVA: TO effect: *F*(1,28) = 24.583, *p* < 0.01; VPA effect: *F*(1,28) = 26.066, *p* < 0.01; TO × VPA effect: *F*(1,28) = 21.419, *p* < 0.01, followed by LSD *post hoc* test]. **(D)** VPA did not alter the number of rearings. Data are presented as the mean ± SEM. *n* = 8. ^∗∗^*p* < 0.01.

The primary symptoms of BTBR mice are thought to include elevated repetitive self-grooming behavior, similar to the VPA-induced ASD model mice. As reported, the BTBR and VPA-exposed mice did display a markedly elevated frequency of self-grooming compared to the saline-treated B6 mice (Figure 5A,C). However, there was a definite drug effect on the self-grooming behavior of the BTBR mice, which was consistent with that of the VPA-exposed mice. Apparently, TO pretreatment lowered the high frequency of self-grooming in the BTBR and VPA-exposed mice (Figure 5A,C). Regarding the number of rearings, there was no definite difference among the four groups in the two kinds of ASD models (Figure 5B,D). In summary, TO pretreatment could effectively improve the self-grooming behavior of the BTBR and B6 mice exposed to VPA in the early postnatal stage.

### Early Postnatal TO Treatment Rescued the Declined Hippocampal Neurogenesis in the DG of BTBR Mice

**FIGURE 6:**
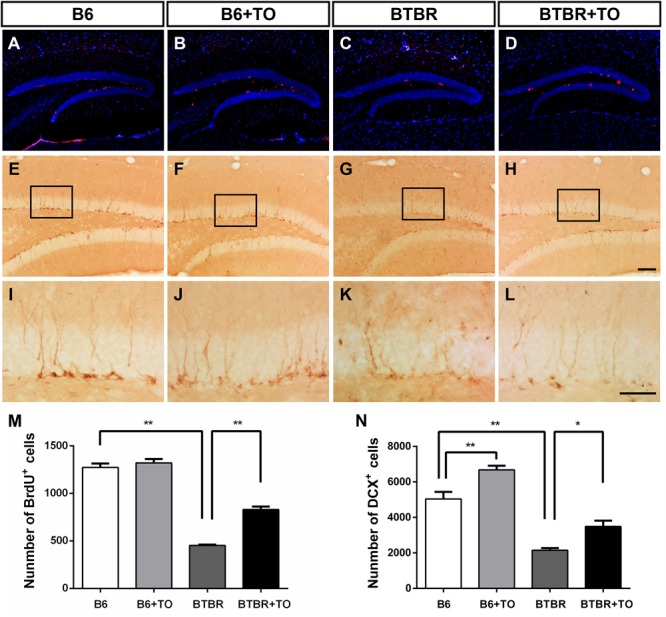
Early postnatal TO treatment promoted an increase in BrdU-labeled cells and DCX-positive cells in the hippocampus. **(A–L)** Representative images of BrdU and DCX immunostaining in the hippocampus of each of the four groups. **(M,N)** As determined with stereological cell quantification of BrdU^+^ [two-way ANOVA: drug effect: *F*(1,8) = 40.833, *p* < 0.01; genotype effect: *F*(1,8) = 391.010, *p* < 0.01; drug × genotype effect: *F*(1,8) = 24.813, *p* < 0.01, followed by LSD *post hoc* test] and DCX^+^ cells [two-way ANOVA: drug effect: *F*(1,8) = 26.544, *p* < 0.01; genotype effect: *F*(1,8) = 109.886, *p* < 0.01; drug × genotype effect: *F*(1,8) = 0.262, *p* > 0.05, followed by LSD *post hoc* test] in the SGZ, BTBR mice had significantly decreased numbers of BrdU^+^ and DCX^+^ cells in the SGZ compared with the SGZ of B6 mice, and TO promoted proliferation of these cells. Data are presented as the mean ± SEM. *n* = 3. Scale bar in **(H)** = 100 μm and applies to **(A–H)**. The scale bar in **(L)** = 50 μm and applies to **(I–L)**. ^∗^*p* < 0.05, ^∗∗^*p* < 0.01.

Cell proliferation was evaluated with BrdU incorporated into the DNA in adult DG neurogenesis and was detected in B6 and BTBR mice with or without early postnatal TO treatment (Figure 6A–D). Specifically, the number of BrdU-labeled cells was significantly decreased in the SGZ of BTBR mice compared with that in B6 mice (Figure 6M). The quantity of these cells in the SGZ of BTBR mice was greatly rescued following TO treatment (Figure 6M), indicating that the inhibition of hippocampal precursor proliferation in the BTBR mice could be improved with early postnatal TO treatment.

In addition, DCX immunostaining is the gold standard for assessing immature neurons (Figure 6E–L). There was a significant reduction of DCX-marked cells in the SGZ of the BTBR mice compared with that of the B6 mice (Figure 6N). However, TO pretreatment could greatly reverse the difference (Figure 6N). In addition, TO pretreatment markedly increased DCX-positive cells in the SGZ of B6 mice (Figure 6N). Taken together, the results showed that early postnatal TO treatment prevented the suppression of hippocampal neurogenesis in the DG of BTBR mice.

### Early Postnatal TO Treatment Rescued VPA-Induced Hippocampal Neurogenesis in the DG of B6 Mice

**FIGURE 7:**
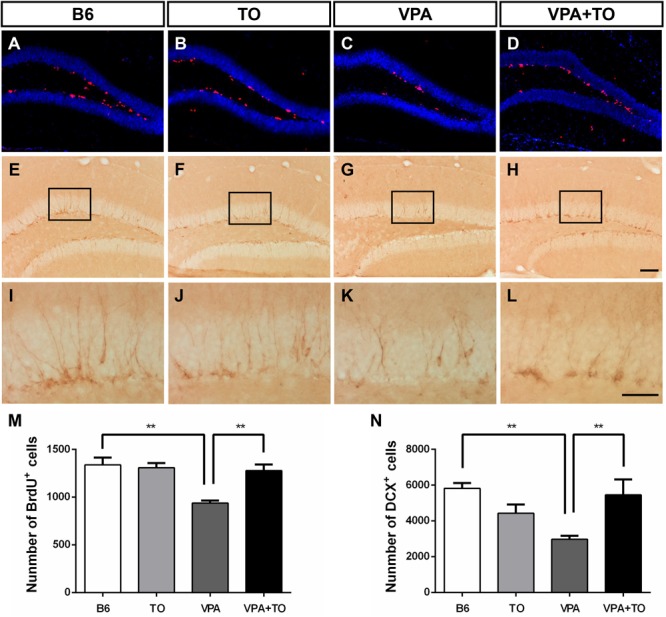
Early postnatal TO treatment prevented the VPA-induced reduction of BrdU^+^ and DCX^+^ cells in the hippocampus. **(A–L)** Representative images of BrdU and DCX immunostaining in the hippocampus of each of the four groups. **(M,N)** As determined with the stereological cell quantification of BrdU^+^ [two-way ANOVA: TO effect: *F*(1,8) = 7.311, *p* < 0.05; VPA effect: *F*(1,8) = 14.067, *p* < 0.01; TO × VPA effect: *F*(1,8) = 10.415, *p* < 0.05, followed by LSD *post hoc* test] and DCX^+^ cells [two-way ANOVA: TO effect: *F*(1,8) = 1.056, *p* > 0.05; VPA effect: *F*(1,8) = 2.977, *p* > 0.05; TO × VPA effect: *F*(1,8) = 13.584, *p* < 0.01, followed by LSD *post hoc* test] in the SGZ, VPA exposure induced a significant reduction of BrdU^+^ and DCX^+^ cells in the SGZ of B6 mice compared with the SGZ of controls, and TO increased the proliferation of these cells. Data are presented as the mean ± SEM. *n* = 3. Scale bar in **(H)** = 100 μm and applies to **(A–H)**. The scale bar in **(L)** = 50 μm and applies to **(I–L)**. ^∗∗^*p* < 0.01.

Currently, VPA exposure during early postnatal development can model ASD, and it is thought to cause a consistent effect on the number of cells incorporated with BrdU or expressing DCX in the DG of VPA-exposed mice (Figure 7A–L). We found that the VPA-exposed mice exhibited impaired hippocampal neurogenesis, characterized by significantly decreased BrdU-labeled cells and DCX-positive immature cells in the SGZ, compared with the B6 mice (Figure 7M,N). The numerical difference in BrdU^+^ and DCX^+^ cells was markedly reversed with TO pretreatment (Figure 7M,N). These results suggested that TO pretreatment ameliorated the VPA-induced impairment of hippocampal neurogenesis.

## Discussion

In the current study, we found that early postnatal TO treatment could significantly improve sociability and reduce repetitive behaviors in two ASD mouse models (i.e., BTBR- and VPA-exposed mice). In addition, the results also showed that the deficits in the hippocampus of these two models could be rescued, as indicated by BrdU and DCX staining. The present study shows that abnormal neurogenesis in the brain may play a critical role in the causes of ASD.

To determine whether TO could activate its target genes, we validated their mRNA levels by means of RT-PCR. TO is a non-selective agonist able to activate both LXRα and LXRβ. However, the data showed that there was little LXRα expression in the cortex and hippocampus of the mice (Whitney et al., 2002). Hippocampus is a classic brain region to study neurogenesis because of its high level of plasticity throughout the lifespan (Xiao et al., 2016; Nam et al., 2017). In addition, BTBR strain, an idiopathic mouse model of autism, and VPA-induced autism model both have impaired hippocampal neurogenesis. Thus, it’s of great value to examine the effect of TO pretreatment on the activation of LXRβ and its target genes, ABCA1 and ABCG1. Our results indicated that the expression of LXRα did not alter after TO administration, but TO could effectively activate LXRβ and its target genes ABCA1 and ABCG1 in the hippocampus of these two mouse models.

The BTBR inbred strain is a common mouse model used in preclinical studies, which shows abnormal social interaction, high levels of repetitive behaviors and reduced ultrasonic vocalizations. To test the impaired sociability of this model, the three-chambered social experiment was applied to our experiment. We found that early postnatal TO administration could significantly alleviate the deficits in the sociability of BTBR mice. In addition, the repetitive self-grooming behavior was also effectively reduced by TO injection. Additionally, we adopted another autistic mouse model, VPA-exposed mice, to validate the therapeutic potential of early postnatal TO treatment and found that TO also contributed to improving the social deficits and repetitive self-grooming behavior of these mice.

Although the exact underlying mechanism of ASD remains unknown, defects in neurogenesis have been shown to be related to this disease both in human and animal models (Wegiel et al., 2010; Bostrom et al., 2016; Gilbert and Man, 2017). Throughout life, active neurogenesis is mainly found in the subventricular zone (SVZ) and SGZ in mammalian brains (Apple et al., 2017). Defects in these regions may have a significant influence on brain functions. MRI evidence showed that there was a significant anatomical abnormality between autistic patients and healthy controls (aged 29 months to 49 years) in the area dentata (AD; DG+CA4) (Saitoh et al., 2001). To date, there is no consensus regarding hippocampal volume. Another clinical study found that the enlargement of the amygdala and hippocampus existed in autistic adolescents (aged 12 to 18 years) compared to the healthy controls (Groen et al., 2010). In addition, an autopsy study revealed multiregional abnormalities in neurogenesis (including DG), neuronal migration and maturation in the ASD brain, which may result in the heterogeneity of the patients’ symptoms (Wegiel et al., 2010).

Recently, several autistic mouse models have shown that there are significant changes in the neurogenesis of the hippocampus. MECP2 transgenic mice display reduced maturation of developing neurons and abnormalities in the adult hippocampus (Chen et al., 2017). A drastic decrease was found in adult neurogenesis in the ventral DG in both Shank3+/ΔC and Cntnap2-/- transgenic mice (Cope et al., 2016). Moreover, evidence also showed that there was reduced neurogenesis in the adult hippocampus of the BTBR and VPA-exposed mouse models (Yochum et al., 2008; Stephenson et al., 2011; Juliandi et al., 2015). These findings suggest that neurogenesis in the hippocampus may be related to behavioral deficits in ASD patients and animal models. Impaired adult hippocampal neurogenesis contributed to not only abnormal sociability but increased stereotyped motor behaviors (Wu et al., 2014; Rusznak et al., 2018). Consistent with previous evidence, our study confirmed that the adult BTBR and VPA-exposed mice displayed a significant decrease in the number of BrdU- and DCX-labeled cells in the hippocampus. However, early postnatal TO treatment could markedly rescue the neurogenesis deficits in these autistic mouse models. It is noteworthy that TO pretreatment increased the DCX- but not BrdU- cells in the SGZ of B6 mice that matched with BTBR mice instead of VPA-induced B6 mice, indicating that different administration methods may lead to not exactly the same effect on hippocampal neurogenesis in two mouse models sharing different pathogenesis of autism. Furthermore, BrdU^+^ cells refer to different kinds of proliferating neurons incorporated with BrdU, which indicates an instantaneous state. However, DCX immunostaining is a gold standard to assess neurogenesis. In view of the heterogeneity of autism, the effects of TO pretreament on social behaviors in two mouse models were also not all the same. These results may indicate that TO can improve the behavioral deficits of the BTBR and VPA-exposed mice by promoting hippocampal neurogenesis.

To date, most preclinical studies on ASD have only tested acute drug treatment effects in adult autistic animal models. Regrettably, there is still little evidence that early intervention is a promising therapy for ASD. It has been identified that deficits in adult behaviors and spine morphology could be rescued by early social enrichment in the Fragile X Syndrome mouse model (Oddi et al., 2015). Another preclinical study indicated that vitamin D treatment could prevent autistic behaviors in the maternal immune activation ASD mouse model during pregnancy (Vuillermot et al., 2017). Our recent study demonstrated that early metformin administration during P7-P14 could rescue the behavioral abnormalities of adult BTBR mice (Wang et al., 2018). Early intervention was also applied to our current study; TO treatment was administered before P14, which approximates the period from the last trimester of pregnancy to the first few postnatal years in humans (Olney et al., 2002). It has been uncovered that our strategy of early intervention did contribute to improvements in the impaired behaviors and the suppressed hippocampal neurogenesis in these two models, which might remind us to pay more attention to early treatment of ASD.

In conclusion, the present study shows that behavioral abnormalities in the above two ASD mouse models can be improved by early postnatal TO injection. The possible mechanism may lie in the effect of TO on restoring suppressed hippocampal neurogenesis, which offers a potential approach for early ASD treatment.

## Data Availability

The raw data supporting the conclusions of this manuscript will be made available by the authors, without undue reservation, to any qualified researcher.

## Ethics Statement

All experimental procedures were approved by the Third Military Medical University and were also performed in accordance with the Guidelines for Animal Care and Use. Every effort was made to restrict the use of animals to as few as possible in our experiments.

## Author Contributions

YC and HZ conducted the experiments, collected and analyzed the data, and drafted the manuscript. XL, RX, and LW contributed to acquisition and analysis of the data. XF designed the experiments, supervised the project, and revised the manuscript.

## Conflict of Interest Statement

The authors declare that the research was conducted in the absence of any commercial or financial relationships that could be construed as a potential conflict of interest.

## Abbreviations

ASD: Autism spectrum disorder
B6: C57BL/6J
BrdU: bromodeoxyuridine
BTBR: BTBR T+ tf/J
DCX: doublecortin
DG: dentate gyrus
DMSO: dimethyl sulfoxide
LXR: liver X receptor
PD: postnatal day
SGZ: subgranular zone
TO: TO901317
VPA: valproic acid
